# Elevated Level of Serum C-reactive Protein Predicts Postoperative Delirium among Patients Receiving Cervical or Lumbar Surgery

**DOI:** 10.1155/2020/5480148

**Published:** 2020-08-10

**Authors:** Quan Ren, Ya-zhou Wen, Jin Wang, Jing Yuan, Xu-hui Chen, Yubaraj Thapa, Meng-shuang Qiang, Fei Xu

**Affiliations:** ^1^Department of Anesthesiology, Zhongda Hospital and School of Medicine, Southeast University, 87 Dingjiaqiao Rd, Nanjing, Jiangsu 210009, China; ^2^Ophthalmology Department, Tongji Hospital, Huazhong University of Science and Technology, 1095 Jiefang Avenue, Wuhan, Hubei 430030, China; ^3^Nanjing Municipal Center for Disease Control and Prevention, 3 Zizhulin, Nanjing, Jiangsu 210003, China

## Abstract

**Objective:**

To explore the relationship between elevated serum C-reactive protein (CRP) level and postoperative delirium (POD).

**Methods:**

206 patients scheduled to receive cervical or lumbar vertebra surgery under general anesthesia for more than 2 hours in a single medical center were observed and analyzed. Patients' serum CRP, delirious status (using the confusion assessment method (CAM)), and delirious score (using the memorial delirium assessment scale (MDAS)) were examined before surgery and 1-2 days after surgery. The association of a serum CRP elevation value from before to after surgery (D-CRP) with delirium occurrence within 2 days after surgery was assessed with a binary logistic regression model, while the association of D-CRP with the postoperative delirious score was assessed with a linear regression model. The effect of D-CRP on predicting delirium occurrence was evaluated with the area under the receiver operating characteristic (ROC) curve (AUC).

**Results:**

D-CRP was significantly positively associated with postoperative delirium occurrence (OR = 1.047, 95%CI = 1.013, 1.082), and D-CRP was also significantly linearly associated with the postoperative delirious score (*β* = 0.014, 95%CI = 0.006, 0.023). AUC of ROC was 0.711 (*P* = 0.014), suggesting that D-CRP had moderate efficacy on predicting postoperative delirium occurrence (*P* < 0.05).

**Conclusions:**

Elevated serum CRP after surgery may be a risk factor for and a predictor of postoperative delirium.

## 1. Introduction

Postoperative delirium (POD) is a common complication of the central nerve system after surgical operations in elderly patients, usually characterized by a fluctuating course of altered consciousness, disordered thinking, and inattention [1]. The incidence of POD is 4% to 65% among adult noncardiovascular surgical patients, 35% to 65% in patients undergoing hip fracture surgery, and 9% to 15% in patients receiving elective orthopedic surgery [2]. Compared to other major complications after major surgeries, delirium occurs more frequently and has a greater significance at the population level [3]. Accumulating evidence suggests that delirium itself might lead to permanent cognitive decline and dementia in some patients [4].

It is well established that surgeries, especially major surgeries, are associated with significant system inflammation. The response of the brain to inflammation, including activation of microglia and neuronal apoptosis, may lead to synaptic and neurochemical disturbances [5]. It has been evidenced that central nervous system inflammation is associated with POD, although the pathogenesis was not fully elucidated [6].

Elevated serum C-reactive protein (CRP) level is widely used to indicate acute systemic inflammation in clinical practices [7–9]. Increased CRP has been reported to be associated with some mental disorders [10, 11]. Two large epidemiological studies found that increased levels of CRP were associated with all dementias [12, 13]. It was even documented that high CRP levels could predict the incidence and recovery of delirium in elderly patients [14]. Moreover, preoperative CRP was examined to be a risk factor for postoperative delirium in a surgical setting [15]. So, it is reasonable to hypothesize that an increase in perioperative CRP level may be associated with the risk of POD for surgical patients. To test this hypothesis, we conducted a hospital-based study to investigate the relationship between increased perioperative serum CRP level and the risk of experiencing POD among patients who received a single type of surgical operation in a Chinese general hospital.

## 2. Methods

### 2.1. Study Design and Participants

**Figure 1:**
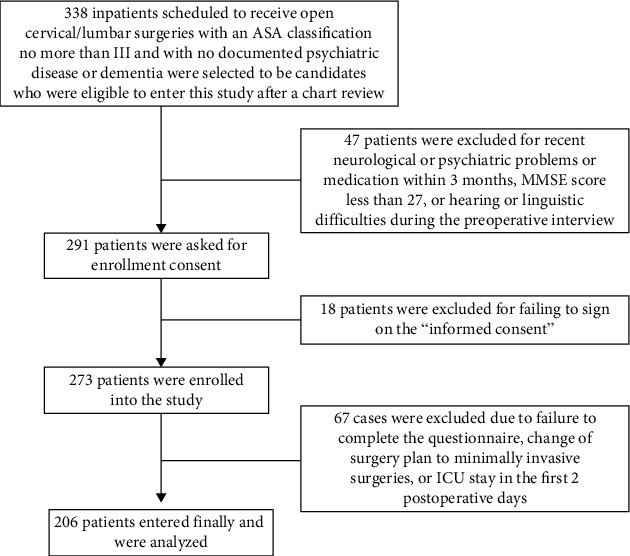
Participant flow diagram.

This study was a prospective observational investigation conducted from June to October in 2014 in Zhongda Hospital of Southeast University, Nanjing, China. Zhongda Hospital is a large general hospital with the top rank in China and admits approximately 18000 patients for surgeries every year. The eligible participants were adult cervical and/or lumbar inpatients if they (1) were scheduled to receive open surgeries (nonminimally invasive surgeries), such as transforaminal lumbar interbody fusion (TLIF), spinal canal enlargement and decompression, anterior or posterior cervical decompression, or posterior approach of scoliosis with internal fixation and fusion; (2) had American Society of Anesthesiologists (ASA) classification not more than III; (3) had no history of neuropsychiatric disease or dementia and had no recent central neurological damage or psychiatric medication within 3 months; (4) had no cognitive impairment, which was assessed in this study using the Chinese Mini-Mental State Examination (MMSE), which includes 19 independent items with a score of 30 in total [16] (patients with a MMSE score less than 27 were considered having cognitive impairment and were not recruited for our study); and (5) had an integrate ability to hear and speak and were willing to be involved in the study. 273 eligible participants were initially recruited. However, those patients who did not complete the questionnaires or were moved to the intensive care unit (ICU) in both postoperative days 1 and 2 were excluded from analysis, thus resulting in 206 patients finally included in the study.  Figure 1 presents the participant flow diagram.

Written informed consent was obtained for each participant. This study was reviewed and approved by the ethics committee of Zhongda Hospital of Southeast University. All identifiable information was removed prior to data analysis.

### 2.2. Anesthesia and Surgery

All patients received no preoperative medication. After entering the operation room, peripheral and central venous access was established and the invasive blood pressure (IBP), electrocardiograph (ECG), central venous pressure (CVP), and pulse oxygen saturation (SpO_2_) were monitored. Midazolam 0.03-0.05 mg/kg (or a loading dose of dexmedetomidine 0.5 *μ*g/kg infusion for 10 minutes, alternatively), sufentanil 0.2-0.3 *μ*g/kg, propofol 1-1.2 mg/kg, and rocuronium bromide 0.6-0.8 mg/kg were injected intravenously in a sequential manner for general anesthesia induction. Endotracheal intubation was performed after adequate muscle relaxation, followed by mechanical ventilation (tidal volume 5-10 mL/kg, ventilation frequency 10-14 times/min, and maintaining P_ET_CO_2_ 30-40 mmHg). Anesthesia was maintained with 1% sevoflurane inhalation and intravenous infusion of propofol 1.5-2.0 mg/kg/h and remifentanil 1-5 *μ*g/kg/h to keep circulation stability. For those who received dexmedetomidine infusion during anesthesia induction, dexmedetomidine infusions were continued at the rate of 0.1-0.2 *μ*g/kg/h during anesthesia maintenance. Bolus cisatracurium 10 mg was applied intermittently when necessary to keep muscle relaxation. In case of hypotension over 20% of the patient's baseline blood pressure, a bolus dose of dopamine 2-3 mg, or bolus atropine 0.25 mg for bradycardia under 50 bpm, was applied for rescue. Infusion speed and infusion type were adjusted referring to CVP and arterial blood gas to maintain the patients' CVP similar to their preoperative baseline values and hemoglobin (HB) above 90 g/L.

The surgeries were cervical or lumbar internal fixation using a steel plate with or without spinal canal decompression. Most of the surgeries were performed under prone position except for some of the cervical surgeries which were performed under supine position and few surgeries which were performed under both supine and prone positions.

### 2.3. Study Variables and Measures

#### 2.3.1. Outcome Variable

The outcome variable of this study was postoperative delirium, which was assessed using the confusion assessment method (CAM), an instrument for qualitatively measuring the status of delirium [3], and the memorial delirium assessment scale (MDAS), a scale to quantitatively estimate delirium severity [4]. POD, as a common postoperative complication, usually occurs between postoperative days 1 and 3 and actually most often on day 1 or day 2 after surgery [17–20]. Moreover, such an approach has been used in previous studies for identifying POD cases in that POD was assessed once on each of day 1 and day 2 after surgery [20–22]. Therefore, we adopted this POD assessment method to identify POD cases in our study. Each participant was assessed regarding status and severity of delirium with CAM and MDAS simultaneously, by a well-trained medical staff who was blinded to the surgical processes, three times at the following time points: 16:00-20:00 (the day before surgery (preoperative)), 14:00-16:00 (the first day after surgery), and 9:00-11:00 (the second day after surgery).

CAM comprises four domains: (a) acute onset and fluctuating course, (b) inattention, (c) disorganized thinking, and (d) change of consciousness level (any state of consciousness beyond complete lucidity, such as alertness, drowsiness, lethargy, or coma). A patient would be regarded as having delirium at the time point, if he/she responded positive to (a), (b), and (c)(/d) at each time of assessment. Otherwise, the patient was regarded as having no delirium at the time point. Subsequently, for analysis, a participant was classified as “yes” to POD if he/she was assessed having POD at any of the 2 postoperative time points. Otherwise, he/she was recorded as “no” to POD in analysis.

MDAS consists of 10 questions about the patients' consciousness level, disorientation, short-term memory impairment, digital span test, disability to maintain attention, disorganized thinking, perception disturbance, delusion, psychomotor activity change, and sleep-wake cycle disturbance. The research member interviewed each patient to assess the potential delirium status and recorded their status as normal (score = 0), mild (score = 1), moderate (score = 2), or severe (score = 3) based on the patient's response to each question. Thus, the potential score sum of MDAS was maximally 30 for a patient at each time point. For analysis, the mean value of scores assessed at two postoperative time points was used for each participant in this study.

#### 2.3.2. Independent Variable

The independent variable of this study was the difference in values of serum CRP between before and after surgery (D-CRP), which was used as the continuous variable. Preoperative and postoperative blood samples of all patients were obtained 1 or 2 days before surgery and 1 day after surgery, respectively. The blood samples were centrifugated for serum and stored in a -80°C refrigerator for further measurement. Serum CRP levels were measured by velocity ratio turbidity (Siemens BN™ II automatic analyzer).

#### 2.3.3. Covariables

Some classical potential confounders have been identified for POD [23]. Therefore, we have several available covariates considered in our study, including age (45 years old, 45-65 years old, or 65+ years old), gender (men or women), ASA grade (I, II, or III classifications), body mass index (BMI) (continuous variable), blood transfusion (categorical variable), anesthesia time (continuous variable), extubation time (continuous variable), and HB (continuous variable).

### 2.4. Data Analysis

Chi-square analysis was used to compare the differences between categorical variables, and Students' *t*-test was used to compare the differences between continuous measures (expressed as mean ± SD). The binary logistic regression model was applied to examine the relationship between D-CRP and postoperative delirium occurrence. The linear regression model was introduced to investigate the relationship between D-CRP and postoperative delirium score. In these two models, the potential confounding factors (age, gender, BMI, blood transfusion, ASA classification, anesthesia duration, extubation time, and postoperative HB value) were adjusted. The area under the ROC curve (AUC) was used to evaluate the predictive effect of D-CRP on POD. *P* < 0.05 was regarded as statistically significant. The software SPSS 22.0 (SPSS Inc., IBM Corporation, Chicago, IL, USA) and Prism 7 (GraphPad, USA) were applied to analyze all the data in this study.

## 3. Results

### 3.1. Selected Characteristics of Participants

Table 1 presents the selected characteristics of participants. Totally, 273 participants were eligible for the study, and finally, 206 patients were included in the study (completion rate = 75.5%). There was no difference in age, gender, BMI, and blood transfusion between those who completed and did not complete the study. For those analyzed participants, their age (mean ± SD) was 57.7 ± 11.3 years old, with a range of 30-85; their BMI (mean ± SD) was 24.5 ± 2.5, with a range of 18.4-32.4; their blood transfusion rate was 30.1%; and 38.8% were men. 12 patients (5.8%) were observed experiencing POD in the first two days after surgical operation. The D-CRP value (mean ± SD) for patients with delirium and those without delirium was 83.9 ± 63.1 and 33.3 ± 31.6 mg/L, respectively (*P* = 0.02). The mean value of the MDAS score was 6.20 ± 3.16 and 0.08 ± 0.39, respectively (*P* < 0.001). There was a significant difference in age, ASA classification, surgery types, mean MDAS score, and postoperative CRP value between those patients having and not having postoperative delirium. However, no difference was observed in gender, BMI, blood transfusion, anesthesia time, extubation time, and postoperative HB between the two groups of participants.

### 3.2. The Relationship between D-CRP and the Risk of POD

Table 2 shows the relationship between D-CRP and the risk of POD occurrence in the studied participants. A significantly positive relationship between D-CRP and POD was observed among adult cervical/lumbar inpatients in this hospital in China. For every 1 mg/L increase in D-CRP, the risk of experiencing POD increased by 2.7%. However, after adjustment for potential confounders (including age, gender, BMI, blood transfusion, ASA classification, anesthesia duration, extubation time, and postoperative HB value), the contribution of D-CRP to POD strengthened in that the odds of experiencing POD would significantly increase by 4.7% for every 1 mg/L increase in D-CRP.

### 3.3. The Correlation between the D-CRP Value and Postoperative Delirium Score

Table 3 displays the correlation between the D-CRP value and postoperative MDAS score among participants in the study. The D-CRP value was examined to be positively correlated with the MDAS postoperative score in this study. For every 1 mg/L increase in D-CRP, the delirium score could be significantly increased by 0.014 unit after adjustment for potential confounding factors.

### 3.4. Predictive Effect of D-CRP on Postoperative Delirium

**Figure 2:**
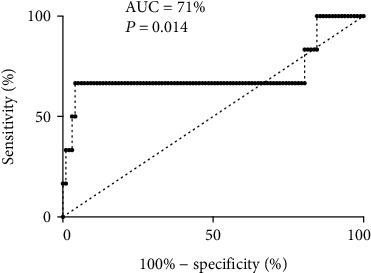
ROC curve of D-CRP with postoperative delirium occurrence.

Based on ROC curve analysis, the AUC was 0.71 (95%CI = 0.50, 0.93) for D-CRP to predict POD occurrence among these study patients, suggesting that D-CRP had a moderate predictive effect on postoperative delirium occurrence among adult cervical/lumbar inpatients (Figure 2).

## 4. Discussion

POD is one of the key manifestations of perioperative neurocognitive disorders (PND) [24]. It is a common early postoperative complication of the central nervous system which often occurs within 7 days after surgery. It is an acute disturbance of nerve function. Its occurrence is highly related to the occurrence of postoperative cognitive dysfunction (POCD), with an increase in mortality [2, 25]. However, the pathogenesis of delirium is not clear to date. Recent studies suggest that delirium is related to the inflammatory response of the central nervous system [25, 26].

CRP is a positive acute-phase protein of inflammation and an objective and reliable indicator of inflammatory response and tissue damage [27]. It is produced by liver cells under the action of proinflammatory factors such as IL-6 and TNF-alpha and reaches the peak within 48 hours [28]. Therefore, the increase in CRP also indicates the increase in IL-6 and TNF-alpha. During systemic inflammation, IL-6, IL-1, IL-1-beta, and TNF-alpha can enter the central nervous system through the blood-brain barrier [29], which can enhance the inflammatory response of the central nervous system, produce reversible and irreversible damage to nerve cells, and cause neurological dysfunction (delirium) or neuron death [25]. Furthermore, high level of serum CRP was reported to increase the blood-brain barrier (BBB) permeability [30], which implicates a decrease in a “barrier” effect of BBB. Therefore, it is speculated that the increase in CRP may indirectly reflect the enhancement of central neuroinflammation and may be a risk factor for delirium requiring pending further investigation. However, the cutoff point of CRP at which the “barrier” effect of BBB would surrender to the systemic inflammation was unknown. In this study, D-CRP was applied to stand for the systemic inflammation enhancement induced from anesthesia and surgery.

There are few reports on the relationship between CRP and postoperative delirium in China. The only published research found online on this issue in the Chinese population reported the predictive effect of high preoperative CRP level on POD [15]. Worldwide, there are several clinical studies that have explored the relationship between CRP and delirium in elderly patients or ICU settings [14, 31–35]. They found that CRP level was highly correlated with delirium occurrence. MacDonald et al. [14] even thought CRP could predict the occurrence and recovery of delirium. The results of this study are consistent with those previous studies, but the previous studies were all reports on elderly (over 70 years old) nonsurgical patients or critical patients, and their basic CRP level and delirium incidence are significantly higher than this study. This study observed a younger population (57.7 ± 11.3 years old) and was aimed at adjusting the confounding factor, old age, and focused on the relationship of CRP elevation amplitude with postoperative delirium occurrence; thus, the results of this study clearly showed the independent influence of surgery and anesthesia on the systemic inflammation and postoperative delirium.

CAM is an internationally recognized method for judging a delirium state, with a sensitivity of 94%-100% and a specificity of 90%-95% [36]. MDAS is a scoring system of delirium severity. It has a high degree of fit with DRS (delirium rating scale) and MMSE which are commonly used in psychiatry to judge the severity of delirium, and it is better than the two [37]. Now, MDAS has been widely used in clinical and research settings. Therefore, we used CAM and MDAS to evaluate the delirium status and the severity of delirium.

The purpose of this study was to explore the relationship between the perioperative increase in CRP and delirium occurrence after operation. In order to reduce the heterogeneity of research objects from a clinical context, a single type of anesthesia and operation was applied in the study. Further, to reduce the influence of possible confounding factors, multivariate analysis models were applied to adjust the factors, such as age, gender, BMI, blood transfusion, and ASA grade. Both logistic and linear regression analyses showed that the increase in CRP after surgery was significantly related to the occurrence of delirium. Moreover, ROC curve analysis showed that the increment amplitude of CRP had a moderate predictive effect on the occurrence of postoperative delirium, which suggested that the increase in CRP after anesthesia and surgery was a risk factor for postoperative delirium occurrence.

The incidence of postoperative delirium (5.8%) among participants in this study was lower than that reported from previous studies, whereas POD incidence ranged from 35% to 65% for cardiac surgery or total hip replacement and 9%-15% for elective orthopedic surgeries [2]. This might be explained by the following reasons. (1) Surgery types differ in our study (cervical and lumbar vertebral surgery) from others (cardiac surgery, hip replacement, or other orthopedic surgeries). (2) The observation time (2 postoperative consecutive days only) was short, and evaluation periods were not consecutive (once in each of the two days only) in this study, which might cause potentially underestimation of delirium cases. (3) It was possible that cases of hypoactive-type delirium might be missed out, thus causing underestimation of the total POD cases. (4) The application of dexmedetomidine in some cases of anesthesia in this study could be another compromise. It is believed that dexmedetomidine could reduce the inflammatory response [38–40] and the incidence of postoperative delirium [41]. And (5) we did not exclude younger patients (under 65 years old) from this study considering that the inflammation response to the same surgical wound could be the same in young people and the elderly. However, old age is an independent risk factor for delirium occurrence [2], which could be another reason of low postoperative delirium incidence in this study.

There are some limitations in this study. First, POD was assessed once daily within day 1 and day 2 after surgery in our study, so such a short observation time might imply that potential POD cases might not be identified if they occurred at different time points in days 1-2 or occurred in days 3-7 after surgery, although evidence has suggested that most POD occurs within the first two postoperative days and POD assessment once daily was acceptable [17–22]. This might lead to underestimation of POD incidence and consequently might reduce the potential statistical power for examining CRP-POD association in our study. Second, the sample size was relatively small, and participants were from one hospital in our study. However, this study is still persuasive. We used the multivariate statistical models to adjust for the possible confounding factors. And the three analysis methods yielded consistent conclusions, which to a more extent showed that there was a correlation between the rise of CRP and the occurrence of delirium. Pending studies are needed to address the role of anti-inflammation factors in postoperative delirium occurrence in surgical settings. Third, there might be a potential participant selection bias in this study. Considering that minimally invasive surgeries would cause much less damage to the tissue, less surgery duration, less bleeding, and less intensity and durance of anesthesia than nonminimally invasive surgeries, we excluded patients who received minimally invasive surgeries in this study. Therefore, it should be prudent to interpret findings from this study. Fourth, we were not able to take all potential confounding factors into account, for example, the Charlson comorbidity index (CCI) score [23], which was reported to be associated with POD incidence. In the future, well-designed large-scale studies will be in need to further explore CRP-POD association with full consideration of POD classical influencing factors under different contexts.

In conclusion, our study suggests that elevated serum level of CRP alone is a risk factor for and predicts postoperative delirium in patients receiving cervical-lumbar surgeries.

## Figures and Tables

**Table 1:** Selected characteristics of 206 participants in this study.

		Participants (206)		*P* value
		Having no delirium (194)	Having delirium (12)	
Age group, *n* (%)	<45	24 (12.4)	0 (0)	0.011
	≥45 and <65	118 (60.8)	4 (33.3)	
	≥65	52 (26.8)	8 (66.7)	
Gender, *n* (%)	Male	78 (40.2)	2 (16.7)	0.133
	Female	116 (59.8)	10 (83.3)	
ASA classification, *n* (%)	I	42 (21.6)	0 (0)	<0.001
	II	150 (77.3)	10 (83.3)	
	III	2 (1.0)	2 (16.7)	
BMI (mean ± SD)		26.5 ± 6.3	25.6 ± 2.1	0.747
Surgery type, *n* (%)	Cervical	30 (15.5)	6 (50)	0.008
	Lumbar	164 (84.5)	6 (50)	
MDAS (mean ± SD)	Preoperative	0.08 ± 0.28	0.33 ± 0.78	0.011
	Postoperative (average)	0.08 ± 0.39	6.20 ± 3.16	0.000
CRP (mg/L, mean ± SD)	Preoperative	4.50 ± 10.85	3.03 ± 2.92	0.641
	Postoperative	37.84 ± 32.37	86.62 ± 62.04	0.000
Length of anesthesia (hours)		3.4 ± 1.06	4.05 ± 1.80	0.051
Extubation time (minutes)		21.2 ± 13.0	17.5 ± 6.0	0.329
Intraoperative blood transfusion, *n* (%)		58 (29.9)	5 (41.7)	0.519
Postoperative HB (g/L)		108.5 ± 18.81	110.4 ± 14.28	0.732

**Table 2:** The relationship between the D-CRP and the risk of postoperative delirium.

		Model 1^#^		Model 2^∗^	
		OR	95% CI	Adj.OR	95% CI
Postoperative delirium	No	1		1	
	Yes	1.027	1.014, 1.041	1.047	1.013, 1.082

^#^Model 1 is a univariate logistic regression analysis. ^∗^Model 2 is a multivariate logistic regression model with adjustment for age, gender, ASA classification, BMI, blood transfusion, duration of anesthesia, extubation time, and postoperative HB.

**Table 3:** The relationship between the D-CRP and postoperative delirium score.

	*β*	95% CI	Standard error	*P* value
Single-factor linear regression model	0.019	0.013, 0.024	0.003	<0.001
Multivariate linear regression model^∗^	0.014	0.006, 0.023	0.004	0.001

^∗^The age, gender, ASA classification, BMI, blood transfusion, duration of anesthesia, extubation time, and postoperative HB were adjusted in the multivariate linear regression model.

## Data Availability

The data used to support the findings of this study are available from the corresponding author upon request.

## References

[1] Aranake-Chrisinger A., Cheng J. Z., Muench M. R. (2018). Ability of postoperative delirium to predict intermediate-term postoperative cognitive function in patients undergoing elective surgery at an academic medical centre: protocol for a prospective cohort study. *BMJ Open*.

[2] Rudolph J. L., Marcantonio E. R. (2011). Postoperative delirium. *Anesthesia & Analgesia*.

[3] Zenilman M. E. (2017). Delirium an important postoperative complication. *JAMA*.

[4] Inouye S. K., Westendorp R. G. J., Saczynski J. S. (2014). Delirium in elderly people. *Lancet*.

[5] Hughes C. G., Patel M. B., Pandharipande P. P. (2012). Pathophysiology of acute brain dysfunction: what's the cause of all this confusion?. *Current Opinion in Critical Care*.

[6] Cerejeira J., Firmino H., Vaz-Serra A., Mukaetova-Ladinska E. B. (2010). The neuroinflammatory hypothesis of delirium. *Acta Neuropathologica*.

[7] Aguiar F. J. B., Ferreira-Júnior M., Sales M. M. (2013). C-reactive protein: clinical applications and proposals for a rational use. *Revista da Associacao Medica Brasileira (1992)*.

[8] Pearson T. A., Mensah G. A., Alexander R. W. (2003). Markers of inflammation and cardiovascular disease: application to clinical and public health practice: a statement for healthcare professionals from the Centers for Disease Control and Prevention and the American Heart Association. *Circulation*.

[9] Windgassen E. B., Funtowicz L., Lunsford T. N., Harris L. A., Mulvagh S. L. (2015). C-reactive protein and high-sensitivity C-reactive protein: an update for clinicians. *Postgraduate Medicine*.

[10] Karlović D., Serretti A., Vrkić N., Martinac M., Marčinko D. (2012). Serum concentrations of CRP, IL-6, TNF-*α* and cortisol in major depressive disorder with melancholic or atypical features. *Psychiatry Research*.

[11] Luan Y. Y., Yao Y. M. (2018). The clinical significance and potential role of C-reactive protein in chronic inflammatory and neurodegenerative diseases. *Frontiers in Immunology*.

[12] Engelhart M. J., Geerlings M. I., Meijer J. (2004). Inflammatory proteins in plasma and the risk of dementia: the Rotterdam Study. *Archives of Neurology*.

[13] Schmidt R., Schmidt H., Curb J. D., Masaki K., White L. R., Launer L. J. (2002). Early inflammation and dementia: a 25-year follow-up of the Honolulu-Asia Aging Study. *Annals of Neurology*.

[14] Macdonald A., Adamis D., Treloar A., Martin F. (2007). C-reactive protein levels predict the incidence of delirium and recovery from it. *Age and Ageing*.

[15] Xiang D., Xing H., Tai H., Xie G. (2017). Preoperative C-reactive protein as a risk factor for postoperative delirium in elderly patients undergoing laparoscopic surgery for colon carcinoma. *BioMed Research International*.

[16] Katzman R., Zhang M., Ouang-Ya-Qu Z. W. (1988). A Chinese version of the Mini-Mental State Examination; impact of illiteracy in a Shanghai dementia survey. *Journal of Clinical Epidemiology*.

[17] Silverstein J. H., Timberger M., Reich D. L., Uysal S. (2007). Central nervous system dysfunction after noncardiac surgery and anesthesia in the elderly. *Anesthesiology*.

[18] Saczynski J. S., Marcantonio E. R., Quach L. (2012). Cognitive trajectories after postoperative delirium. *The New England Journal of Medicine*.

[19] Lee H., Oh S. Y., Yu J. H., Kim J., Yoon S., Ryu H. G. (2018). Risk factors of postoperative delirium in the intensive care unit after liver transplantation. *World Journal of Surgery*.

[20] Whitlock E. L., Vannucci A., Avidan M. S. (2011). Postoperative delirium. *Minerva Anestesiologica*.

[21] Dong R., Sun L., Lu Y., Yang X., Peng M., Zhang Z. (2017). NeurimmiRs and postoperative delirium in elderly patients undergoing total hip/knee replacement: a pilot study. *Frontiers in Aging Neuroscience*.

[22] Shi Z., Mei X., Li C. (2019). Postoperative delirium is associated with long-term decline in activities of daily living. *Anesthesiology*.

[23] Chu C. S., Liang C. K., Chou M. Y. (2016). Short-Form Mini Nutritional Assessment as a useful method of predicting the development of postoperative delirium in elderly patients undergoing orthopedic surgery. *General Hospital Psychiatry*.

[24] Subramaniyan S., Terrando N. (2019). Neuroinflammation and perioperative neurocognitive disorders. *Anesthesia and Analgesia*.

[25] Cunningham C. (2011). Systemic inflammation and delirium: important co-factors in the progression of dementia. *Biochemical Society Transactions*.

[26] Cerejeira J., Lagarto L., Mukaetova-Ladinska E. B. (2014). The immunology of delirium. *Neuroimmunomodulation*.

[27] Pinato D. J., Bains J., Irkulla S. (2013). Advanced age influences the dynamic changes in circulating C-reactive protein following injury. *Journal of Clinical Pathology*.

[28] Pepys M. B., Hirschfield G. M. (2003). C-reactive protein: a critical update. *The Journal of Clinical Investigation*.

[29] Banks W. A., Kastin A. J., Gutierrez E. G. (1994). Penetration of interleukin-6 across the murine blood-brain barrier. *Neuroscience Letters*.

[30] Hsuchou H., Kastin A. J., Mishra P. K., Pan W. (2012). C-reactive protein increases BBB permeability: implications for obesity and neuroinflammation. *Cellular Physiology and Biochemistry: International Journal of Experimental Cellular Physiology, Biochemistry, and Pharmacology*.

[31] White S., Eeles E., O'Mahony S., Bayer A. (2008). Delirium and C-reactive protein. *Age and Ageing*.

[32] Speciale S., Bellelli G., Guerini F., Trabucchi M. (2008). C-reactive protein levels and delirium in a rehabilitation ward. *Age and Ageing*.

[33] Morandi A., Sleiman I., Rozzini R., Trabucchi M. (2007). C-reactive protein and delirium in acute ill elderly patients. *Age and Ageing*.

[34] George J., Mukaetova-Ladinska E. B. (2007). Delirium and C-reactive protein. *Age and Ageing*.

[35] Zhang Z., Pan L., Deng H., Ni H., Xu X. (2014). Prediction of delirium in critically ill patients with elevated C-reactive protein. *Journal of Critical Care*.

[36] Inouye S. K., van Dyck C. H., Alessi C. A., Balkin S., Siegal A. P., Horwitz R. I. (1990). Clarifying confusion: the confusion assessment method. *Annals of Internal Medicine*.

[37] Breitbart W., Rosenfeld B., Roth A., Smith M. J., Cohen K., Passik S. (1997). The memorial delirium assessment scale. *Journal of Pain and Symptom Management*.

[38] Peng J., Zhang P., Zheng H., Ren Y. Q., Yan H. (2019). Dexmedetomidine reduces hippocampal microglia inflammatory response induced by surgical injury through inhibiting NLRP3. *Chinese journal of traumatology = Zhonghua chuang shang za zhi*.

[39] Tan F., Gan X., Deng Y. (2018). Intraoperative dexmedetomidine attenuates postoperative systemic inflammatory response syndrome in patients who underwent percutaneous nephrolithotomy: a retrospective cohort study. *Therapeutics and Clinical Risk Management*.

[40] Zhang J., Wang Z., Wang Y., Zhou G., Li H. (2015). The effect of dexmedetomidine on inflammatory response of septic rats. *BMC Anesthesiology*.

[41] Duan X., Coburn M., Rossaint R., Sanders R. D., Waesberghe J. V., Kowark A. (2018). Efficacy of perioperative dexmedetomidine on postoperative delirium: systematic review and meta-analysis with trial sequential analysis of randomised controlled trials. *British Journal of Anaesthesia*.

